# Different lasers reveal different skin microcirculatory flowmotion - data from the wavelet transform analysis of human hindlimb perfusion

**DOI:** 10.1038/s41598-019-53213-2

**Published:** 2019-11-18

**Authors:** L. Monteiro Rodrigues, Clemente Rocha, Hugo Ferreira, Henrique Silva

**Affiliations:** 1 CBIOS — Universidade Lusófona’s Research Center for Biosciences and Health Technologies, Av Campo Grande, 1749 024 Lisboa, Portugal; 2 Pharmacol. Sc Depart — Universidade de Lisboa, Faculty of Pharmacy, Av Prof Gama Pinto, 1649 003 Lisboa, Portugal; 3 IBEB — Biophysics and Biomedical Engineering Institute, Universidade de Lisboa Faculty of Sciences, Campo Grande, 1749 016 Lisboa, Portugal

**Keywords:** Computational biophysics, Diagnostic markers

## Abstract

Laser Doppler flowmetry (LDF) and reflection photoplethysmography (PPG) are standard technologies to access microcirculatory function *in vivo*. However, different light frequencies mean different interaction with tissues, such that LDF and PPG flowmotion curves might have distinct meanings, particularly during adaptative (homeostatic) processes. Therefore, we analyzed LDF and PPG perfusion signals obtained in response to opposite challenges. Young healthy volunteers, both sexes, were assigned to Group 1 (n = 29), submitted to a normalized Swedish massage procedure in one lower limb, increasing perfusion, or Group 2 (n = 14), submitted to a hyperoxia challenge test, decreasing perfusion. LDF (Periflux 5000) and PPG (PLUX-Biosignals) green light sensors applied distally on both lower limbs recorded perfusion changes for each experimental protocol. Both techniques detected the perfusion increase with massage, and the perfusion decrease with hyperoxia, in both limbs. Further analysis with the wavelet transform (WT) revealed better depth-related discriminative ability for PPG (more superficial, less blood sampling) compared with LDF in both challenges. Spectral amplitude profiles consistently demonstrated better sensitivity for LDF, especially regarding the lowest frequency components. Strong correlations between components were not found. Therefore, LDF and PPG flowmotion curves are not equivalent, a relevant finding to better study microcirculatory physiology.

## Introduction

Laser Doppler flowmetry (LDF) and imaging (LDI) are the gold standard technologies in vascular medicine, although high cost (including operation-time cost), oversensitivity, and multiple difficulties in data interpretation limit their usefulness^1–3^. Photoplethysmography (PPG**)** is also a practical reference, also non-invasive, made popular by its low cost (acquisition and operation) and portability^4–7^. Better sensors and more effective wireless communications have renewed its interest such that PPG is currently no longer only an alternative to electrocardiography (ECG) to measure heart rate, but rather an innovative breakthrough in the wearable health monitoring technologies era, with multiple applications in sight^7–10^.

These two technologies, LDF and PPG, are based in similar biophysics but use different laser frequencies, such that the respective perfusion related information is obtained at different depths, resulting in different discrimination capacities. The perfusion estimation provided by LDF is assumed to be linearly related to the velocity and concentration of moving erythrocytes^11–13^. Our instrument uses a probe with a standard fiber separation (0.25 mm) and a 780 nm wavelength. The typical measurement depth, according to the manufacturer, is 0.3–0.5 millimeter, with a portion of the signal able to penetrate as deep as 1 mm^1,13,14^. For reflection PPG this depth discrimination depends on the light wavelength used (green and red being the most common) and the distance between the light source and the photo detector^15–17^. Some studies have conducted depth discrimination only based on wavelength^18,19^ while others were based on both wavelength and source-to-detector distance^2^. A recent paper demonstrated that the penetration depth increases with the wavelength of light used on skin^14^. The same computational simulation used by these authors suggests that increasing the beam width also increases the light penetration depth.

The green (530 nm) wavelength light is one of the most frequent choices as it is very resistant to motion related artifacts. It is also considered appropriate for measurements of superficial (0.2–0.4 mm) perfusion^1,2^. However, it is a challenge to ensure the signal is arterial in origin as to identify all other hemodynamic influencers^4,20,21^.

Pulse wave analysis is very susceptible to motion artifacts, such that the Fast Fourier (FF) or the Wavelet Transform (WT) has been used to decompose^22,23^ and “de-noise”^8,24,25^ the spectra of these complex oscillatory signals. The application of WT to the PPG signal led researchers in the field to propose the “augmentation index (AI)” and the “b/a ratio,” which seem to help characterize vascular ageing^26,27^. More recently, the second derivative wave of the original PPG signal (accelerometer data) was referred to contain important health-related information^8,25^.

The true significance of LDF and PPG spectra are important issues from the mechanistic point of view, especially since PPG is often referred as a low-cost replacement for LDF. In previous studies, WT analysis of PPG signals revealed an oscillatory pattern (flowmotion) close to that of LDF^3,28^. A pilot study has shown a good correlation between PPG and LDF for specific physiological frequency bands^29^.

**Figure 1:**
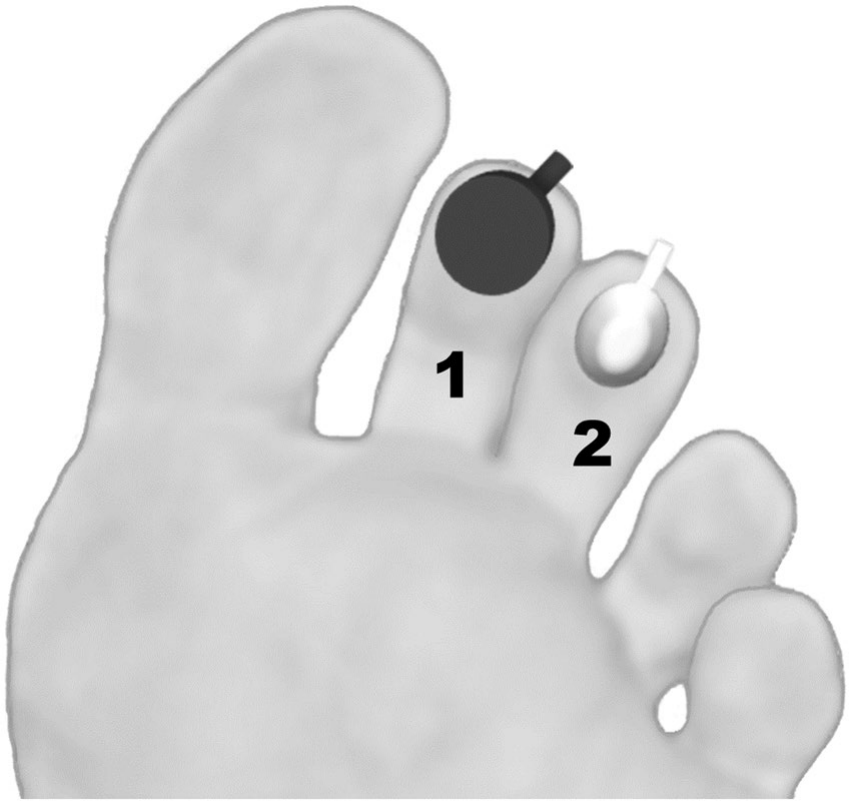
Schematic diagram of the experimental setup to illustrate the application of LDF (1) and PPG (2) probes for the distal assessment of perfusion changes in the human lower limb (see text).

In the present study, we are trying to establish how equivalent are the flowmotion patterns of LDF and PPG signals distally obtained (Fig. 1) in healthy human volunteers, under the same reproducible experimental conditions. Two well-known opposite challengers were applied – a standard (Swedish) massage procedure, known to increase perfusion^30,31^, and hyperoxia, which evokes a decrease in perfusion^28,32^. LDF and PPG signals were followed in all phases of the evoked adaptative responses to each challenge and analyzed by WT. This approach, comparing two gold standards used in vascular medicine instrumentation, as close as possible from the normal physiological state, are meant to help develop more precise approaches to quantitatively assess microcirculatory function, a crucial step to improve patient prognosis^33,34^.

## Results

**Figure 2:**
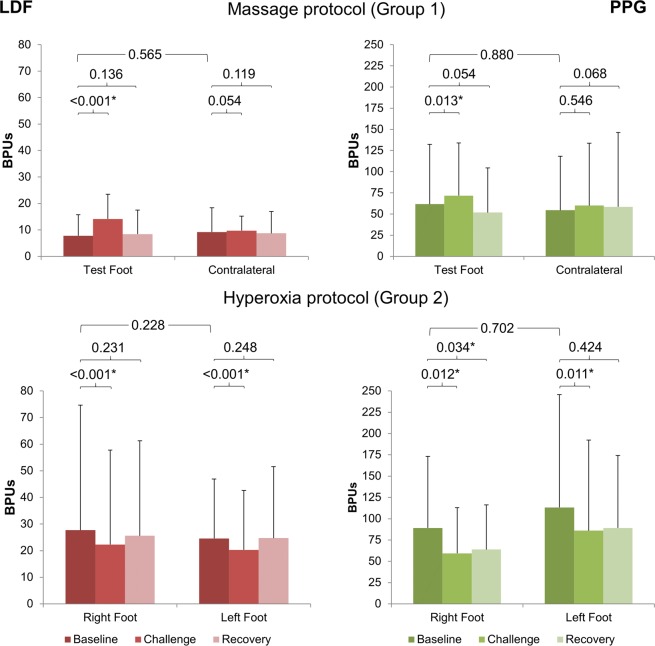
Graphic representation of the evolution of LDF and PPG perfusion changes detected in Group 1 (n = 29) and Group 2 (n = 14) in the distal lower limb, as a result of two opposite vasoactive challengers – massage, known to improve perfusion in the lower limb, and hyperoxia, known to evoke a perfusion reduction. Results (X mean and sd standard deviation) are shown for both limbs. Statistical comparisons between both feet at baseline top line, Mann-Whitney test) and between phases in each foot (Wilcoxon test) are also shown (*p < 0.05).

Perfusion changes obtained during the experimental protocols developed under these two approaches (massage and hyperoxia) are displayed in Fig. 2. Some perfusion differences between limbs are immediately noted in Phase I, with LDF as with PPG, no matter the group. Such differences have been previously reported^35–37^ in assessments made by an optical probe, and have been attributed to the existing spatial vascular variability between feet.

Comparison of all limbs in all individuals (Wilcoxon sign rank test) revealed no significant differences in perfusion (data not shown). Comparing all limb pairs in each group (Mann-Whitney U test) confirmed this finding (Fig. 2).

**Figure 3:**
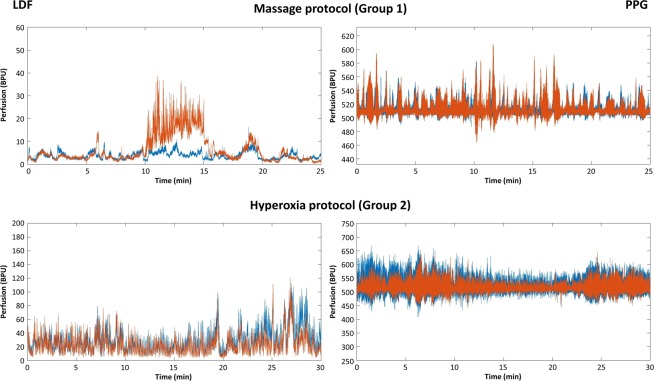
Illustrative examples of LDF (left column) and PPG (right column) obtained in a representative subject. Both limbs are represented in distinct colors – in Group 1 the massage limb is orange and the contralateral is blue; in Group 2 left limb is orange and right limb is blue. As illustrated, massage evokes a perfusion increase detected in both limbs in Phase 2, more obvious in the massaged limb with LDF. Hyperoxia evokes a perfusion decrease also in both limbs in Phase 2, clearly visible with both techniques (see text).

These two challenges induce opposite responses (Fig. 3). Massage increases perfusion in Phase II of the massaged limb but also in the contralateral limb (as discussed ahead). In the test foot, this perfusion increase is statistically significant both with LDF (p < 0.001) and PPG (p = 0.013). On the other hand, hyperoxia evokes a significant decrease of perfusion in Phase II in both limbs, detected with both technologies (LDF p < 0.001 on right and left feet; PPG p < 0.05 on right and left feet). The intense perfusion reduction is not fully recovered in Phase III in all individuals (Figs 2 and 3).

**Figure 4:**
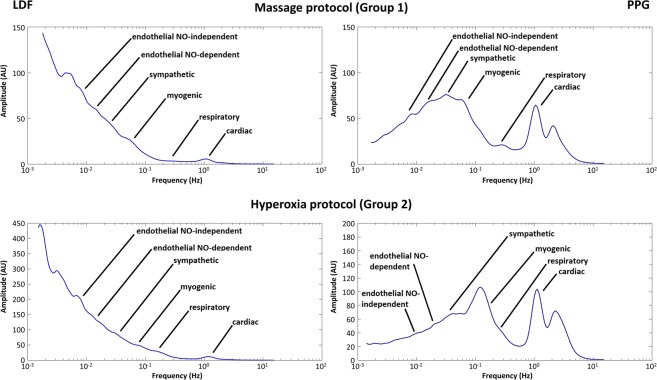
Frequency spectra showing the mean amplitude profiles changes evoked by massage and hyperoxia in LDF (left column) and PPG (right column) (n = 43, data from both limbs). As shown, the lowest frequency range components show wider amplitudes for LDF, determining this characteristic profile which likely results from the higher sensitivity and specificity attributed to this technology. Regarding PPG, wider variations are noted with higher frequency components, primarily cardiac and myogenic (see text).

Examining each signal components’ evolution in response to different challengers (Table 1, Fig. 4), the effects of massage on LDF show a significant increase in the cardiac (p < 0.001), respiratory (p < 0.001) and myogenic (p = 0.016) components during Phase II, compared to baseline. These differences persist in Phase III for the cardiac and respiratory components (p = 0.016 and p = 0.012, respectively). PPG components show a similar significant increase in the cardiac (p = 0.003) and respiratory (p < 0.001) components, and furthermore, a significant decrease in the sympathetic (p < 0.001), NOd (p < 0.001) and NOi (p < 0.001) components. The cardiac (p = 0.010), myogenic (p < 0.001) and sympathetic (p < 0.001) components of PPG signal show significant differences in Phase III compared with baseline.

**Table 1:** LDF and PPG spectral component changes resulting from the application of massage and hyperoxia to Group 1 (n = 29) and Group 2 (n = 14) obtained by the Wavelet Transform (see text).

Component		LDF signal			PPG signal		
		Baseline	Challenge	Recovery	Baseline	Challenge	Recovery
**Massage protocol (Group 1)**							
*Cardiac*	*X*	8.2	11.5	8.3	10.4	12.0	9.7
	*sd*	8.3	7.9	8.4	6.3	4.2	5.2
	*p*	—	<0.001*	0.016*	—	0.003*	0.010*
*Respiratory*	*X*	1.2	1.7	1.3	6.1	18.5	6.8
	*sd*	0.5	0.7	0.7	2.9	8.0	4.1
	*p*	—	<0.001*	0.012*	—	<0.001*	0.736
*Myogenic*	*X*	3.5	5.4	4.0	21.5	22.6	23.8
	*sd*	1.4	2.8	2.1	8.0	6.4	7.4
	*p*	—	0.016*	0.092	—	0.052	<0.001*
*Sympathetic*	*X*	7.7	8.4	8.5	16.3	12.5	14.5
	*sd*	3.6	4.0	4.1	4.1	3.9	3.5
	*p*	—	0.136	0.853	—	<0.001*	<0.001*
*Endothelial NOd*	*X*	13.8	12.8	14.0	17.8	13.6	18.2
	*sd*	5.3	5.7	5.2	6.1	4.1	6.4
	*p*	—	0.128	0.877	—	<0.001*	0.187
*Endothelial NOi*	*X*	23.0	21.6	21.7	13.7	9.6	12.0
	*sd*	5.6	6.6	5.9	4.8	3.3	4.5
	*p*	—	0.082	0.088	—	<0.001*	0.068
**Hyperoxia protocol (Group 2)**							
*Cardiac*	*X*	1.1	1.2	1.1	18.4	15.8	15.9
	*sd*	0.7	0.5	0.4	9.2	6.5	8.2
	*p*	—	0.085	0.866	—	0.095	0.045*
*Respiratory*	*X*	3.0	3.5	2.8	9.5	13.1	11.0
	*sd*	1.9	1.9	1.3	3.2	4.3	4.1
	*p*	—	0.123	0.442	—	<0.001*	0.009*
*Myogenic*	*X*	9.0	8.9	9.3	22.6	25.1	22.7
	*sd*	4.1	4.4	4.3	8.2	8.5	7.2
	*p*	—	0.309	0.952	—	0.013*	0.871
*Sympathetic*	*X*	8.0	6.5	7.9	15.5	13.7	16.1
	*sd*	3.7	2.2	3.0	4.8	4.1	4.7
	*p*	—	0.017*	0.544	—	0.011*	0.114
*Endothelial NOd*	*X*	20.1	18.6	19.4	11.4	9.6	11.2
	*sd*	6.4	6.4	7.5	3.5	3.0	3.6
	*p*	—	0.102	0.321	—	0.012*	0.891
*Endothelial NOi*	*X*	12.8	12.0	14.2	9.6	10.4	11.1
	*sd*	4.3	5.7	4.1	4.9	3.7	4.1
	*p*	—	0.278	0.023*	—	0.993	0.212

Challenge and recovery p-values result from each phase comparisons with baseline (X mean, sd standard deviation, *p < 0.05).

Regarding hyperoxia, LDF cardiac and respiratory components increased in Phase II, but no statistical significance was found when compared with baseline. The myogenic, sympathetic, NOd, and NOi activities decreased during challenge, but only the sympathetic was significantly different (p = 0.017) regarding Phase I. During recovery some differences were still observed, but only NOi was significantly higher (p = 0.023) than baseline. PPG signal components during hyperoxia show a decrease of cardiac (non-significant), sympathetic, and NOd activities (p = 0.011 and p = 0.012, respectively) while the respiratory and myogenic activities increased significantly (p < 0.001 and p = 0.013, respectively). During Phase III all components’ activities returned to baseline except for the cardiac and respiratory, still showing significant differences (p = 0.045 and p = 0.009, respectively) to baseline. In Group 1, positive correlations for LDF and PPG components were found for the cardiac (R = 0.589, p < 0.001), respiratory (R = 0.562, < 0.001), and myogenic (R = 0.279, p = 0.034) components (Table 2). For Group 2 positive correlations could only be found for NOd (R = 0.388, p = 0.023) and NOi (R = 0.378, p = 0.028) components.

**Table 2:** Spearman’s R coefficient for the linear correlation of the perfusion percent change (provocation-baseline) for both LDF and PPG techniques in both protocols (*p < 0.05).

Component		Massage (Group 1)	Hyperoxia (Group 2)
*Cardiac*	R	0.589	−0.093
	*p*	<0.001*	0.599
*Respiratory*	R	0.562	0.057
	*p*	<0.001*	0.750
*Myogenic*	R	0.279	0.134
	*p*	0.034*	0.451
*Sympathetic*	R	0.066	0.278
	*p*	0.622	0.112
*Endothelial NOd*	R	0.127	0.388
	*p*	0.341	0.023*
*Endothelial NOi*	R	0.172	0.378
	*p*	0.197	0.028*

Kurtosis and skewness were also calculated as statistical measures of LDF and PPG spectral distributions (Table 3). Kurtosis of the normal distribution as a value of 3.0. Kurtosis lower than 3.0, also called platykurtic, means the distribution produces fewer and less extreme outliers than does the normal distribution, e.g., low and broad central peaks and short and thin tails. Skewness is a measure of the symmetry in a distribution and essentially measures the relative size of the two tails. A normal distribution will have a skewness of 0 although it may increase depending on the sample size.

**Table 3:** Mean (SD) kurtosis (KT) and skewness (SK) of the LDF and PPG signal spectral bands obtained in both groups.

Component		Massage (Group 1)		Hyperoxia (Group 2)	
		KT	SK	KT	SK
*cardiac*	LDF	1.82 (0.26)	0.13 (0.08)	1.92 (0.20)	0.30 (0.13)
	PPG	1.74 (0.12)	0.17 (0.07)	1.90 (0.50)	0.40 (0.30)
	p	<0.001*	0.001*	0.054	0.040*
*respiratory*	LDF	4.17 (1.06)	1.10 (0.41)	2.95 (0.76)	0.66 (0.35)
	PPG	4.03 (0.87)	1.12 (0.30)	2.80 (0.60)	0.60 (0.30)
	p	0.217	0.866	0.300	0.251
*myogenic*	LDF	2.19 (0.34)	0.18 (0.17)	2.46 (0.45)	0.41 (0.26)
	PPG	2.15 (0.18)	0.10 (0.16)	2.30 (0.20)	0.30 (0.20)
	p	0.095	0.012*	0.001*	0.016*
*sympathetic*	LDF	2.17 (0.30)	0.07 (0.18)	2.19 (0.24)	0.04 (0.24)
	PPG	2.12 (0.24)	0.03 (0.23)	2.10 (0.20)	−0.10 (0.20)
	p	0.007*	0.384	0.003*	0.050*
*endothelial NOd*	LDF	2.17 (0.37)	0.03 (0.22)	2.17 (0.36)	0.03 (0.32)
	PPG	2.16 (0.31)	−0.01 (0.26)	2.10 (0.30)	0.00 (0.20)
	p	0.407	0.401	0.375	0.315
*endothelial NOi*	LDF	2.11 (0.38)	−0.11 (0.31)	2.07 (0.34)	−0.06 (0.32)
	PPG	2.25 (0.42)	0.01 (0.33)	2.20 (0.50)	−0.100 (0.30)
	p	0.125	0.061	0.510	0.244

Statistical comparison between feet and between techniques is presented (T – test foot, C – control foot, *p < 0.05).

Kurtosis in Group 1 was found to be lower than 3.0 for all LDF and PPG components except respiratory (Table 3). LDF components’ kurtosis were higher than PPG except for the NOi. The cardiac and sympathetic kurtosis were significantly higher in LDF (p < 0.001 and p = 0.007) and a positive skewness was present except for NOd in PPG and NOi in LDF, showing negative values. Cardiac component skewness was found to be significantly higher in PPG, while the myogenic skewness was significantly higher with LDF. In Group 2, all LDF and PPG components exhibited a kurtosis lower than 3.0 with higher values for LDF, except for the NOi, and significant differences in the myogenic and sympathetic components (p = 0.001 and p = 0.003, respectively). In turn, skewness profiles were highly variable. The cardiac, respiratory, myogenic, and NOd components had skewnesses > 0 with both LDF and PPG. The sympathetic component had skewnesses > 0 with LDF and a skewnesses < 0 with PPG. The NOi had a skewnesses < 0 with both techniques. The cardiac skewness was significantly lower with LDF (p = 0.040), while the myogenic and sympathetic were significantly higher (p = 0.016 and p = 0.050) with LDF.

## Discussion

LDF and PPG share a common biophysical basis. These techniques produce multi-scaled, non-linear, oscillatory signals, integrating several related physiological influences (cardiac, respiratory, autonomic, among others) that result in a periodic oscillatory pattern also known as the skin (microcirculatory) flowmotion^38–40^. The recognized susceptibility to artifacts (movement, environmental noise, light, etc.), partially explains the multiple difficulties in its interpretation (Fig. 3). LDF uses a laser beam that penetrates the skin producing the “Doppler effect,” which has been long known^11,41^ and is extensively applied to vascular research. Its pulsatile nature, synchronous with the heartbeat together with other respiratory, myogenic, sympathetic, and endothelial contributions, explains these oscillations that produce this non-continuous signal^36,40,42^. Reflection PPG uses visible light to measure the volumetric variations of blood circulation and is composed of pulsatile (AC) and superimposed (DC) components^5^. The AC component is related with the blood (believed to be mostly arterial) volume synchronous variations resulting from the heartbeat which, in turn, relates with the cardiac events^20,43^. The DC component is determined by respiration, autonomic nervous system activity, and thermoregulation^4,5^. Pulse Rate Variability (PRV), extracted from PPG signal, has been proposed to relate with autonomic activity, showing a promising potential to follow up cardiovascular pathophysiology^6,44^. In the present paper we are measuring perfusion changes at different depths, with opposite challengers and under controlled conditions, expecting to find different flowmotion expressions at different depths.

Both technologies detected the perfusion increase following massage, also in the contralateral non-massaged limb, and the perfusion decrease, again in both limbs, following hyperoxia (Fig. 2). These dual (both limbs) observations were originally reported elsewhere, in human as in mice^29,30,32^. This research indicates that LDF and PPG measure perfusion directly, even at different tissue levels.

In order to look deeper into LDF and PPG flowmotion, we analyzed changes occurring under challenge by the wavelet transform (WT), a well-known analytical instrument mostly applied to LDF analysis refinement. For LDF it is accepted that those oscillations, cardiac (0.6–2.0 Hz), respiration (0.15–0.6 Hz), myogenic (∼0.05–0.15 Hz), neurogenic (∼0.02–0.05 Hz), and endothelial (∼0.0095–0.02 Hz), determining skin flowmotion, represent the influence of heart rate, respiration, myogenic^36,42^, autonomical^39,45^, and endothelial dependent and independent vascular smooth muscle relaxation^35,40,46^.

Knowing that PPG signals are obtained at a different wavelength, the same rationale was applied to PPG signals comprised of common pulsatile (e.g. cardiac) and superimposed (e.g. respiratory) components.

As mentioned previously, WT has been used as a de-noising tool to remove Gaussian-like noise from the PPG signal^23,24^, increasing the interest and utility of this pulse waveform. Pulse wave analysis is being currently used to study diabetes, atherosclerosis and cardiovascular disease^8,24,27^, far beyond the original oxygen saturation and heart rate determination.

Our approach is in line with this recent effort to further investigate new information and applications for the PPG signal^8^. LDF and PPG components ranges were found to overlap in cardiac (1.7–0.7 Hz), respiratory (0.7–0.2 Hz), myogenic (0.2–0.05 Hz), sympathetic (0.05–0.027 Hz), endothelial NO-dependent (NOd) (0.027–0.013 Hz), and endothelial NO-independent (NOi) (0.013–0.0064 Hz) ranges, as published previously^28,47^. Table 1 depicts some differences that seem to result directly from the different depth discriminative capacities of LDF and PPG. The few hundred micrometer (<1 mm) depth assumed for LDF and PPG means that both technologies provide estimations in a small superficial vascular volume^1,12,13^ where the high frequency components are still the main determinants of flowmotion. However, the superficial mobilization of blood seems to affect PPG penetration. It is known that in compressed tissues, light penetrates more deeply into the tissue, reaching deeper and larger vessels, which increases PPG signal^8^. Deeper penetration allows access to higher volumes of blood perfusing the area perfusion. For Group 1, LDF only detects significant changes in the high frequency components. However, PPG consistently detects all component changes in the expected direction. For Group 2 the reduced perfusion decomposition observed as a result of central and peripheral interactions of hyperoxia is unevenly described by LDF and PPG. In fact, the high variability from the LDF signal only allows a significant detection for the sympathetic activity reduction, while PPG gives a detailed description for all components according with our expectations (Table 1). Contrary to previously published work^47^, correlations between components from LDF and PPG changes are not strong (Table 2). For Group 1, significant positive correlations are found for the high-frequency cardiac, respiratory, and myogenic components. For Group 2, significant positive correlations are found only for the low-frequency endothelial components.

Analysis of the complete (25 minutes) frequency spectra of LDF and PPG depicts another important feature. Figure 4, representing the mean spectra of all subjects, shows different amplitude profiles for both signals, irrespective of the challenge. As shown, for LDF higher amplitude changes mostly occur in the lowest frequency regions, with myogenic, sympathetic, and endothelial components designing this descendent profile. In contrast, PPG amplitudes vary mostly in the higher amplitude ranges with major contributions from cardiac and myogenic components (high respiratory variability) and much more discrete contributions from the lower frequencies range, especially for Group 2. These observations complement our view resulting, in our opinion, from the high sensitivity of LDF regarding PPG. Although not extensively documented, a few studies have compared sensitivity, specificity, and reproducibility of this technique with others, suggesting that LDF should be regarded as a reference for tissue vitality testing^48,49^. Again, this is in opposition to recent remarks suggesting that PPG could replace LDF for endothelial studies^47^. We should stress that our protocol practically eliminated main variability sources, namely by registering LDF and PPG signals from both limbs^32^, reinforcing the robustness of our data and analysis.

Finally, kurtosis was generally higher in LDF than in PPG spectral bands, especially for high frequency components, suggesting less power distribution in each frequency range (Table 3). Globally, no matter the protocol, differences in kurtosis and skewness were observed more frequently for the myogenic, cardiac, and sympathetic components, with the respiratory and endothelial components consistently showing no differences.

Globally, LDF and PPG seem to be composed of different hemodynamic oscillations, showing different sensitivities to these flowmotion components. The simultaneous use of LDF and PPG seems to complement and detail our view on microcirculatory flowmotion.

## Methods

### Subjects

A convenience set of 43 healthy individuals, both sexes (20.0 ± 2.0 y.o.) was gathered according to previously defined inclusion and non-inclusion criteria. Selected subjects had no relevant clinical history, were non-smokers, not taking any vasoactive medication, and were asked to refrain from consuming caffeine containing beverages 24 hours prior to their participation in the experimental procedures. Female subjects were at different phases of their fertility cycle and not taking oral contraception. All individuals gave their written informed consent prior to the beginning of the study. Two experimental groups were defined and allocated to two perfusion challenge procedures. Group 1 included 29 subjects (19.9 ± 1.6 y.o., 13 males, 16 females) submitted to a massage protocol and Group 2 included 14 subjects (20.3 ± 2.9 y.o., 6 males, 8 females) submitted to a hyperoxia challenge test. Specific conditions to observe in each protocol have been published elsewhere^3,30,37^.

The experimental protocol was previously approved by the Universidade Lusófona’s School of Health Sciences Ethical Commission (code reference: CE03/2013.12) and carried out in accordance with the Declaration of Helsinki and respective amendments, observing good clinical practice for medical research Involving human subjects^50^.

### Experimental

Before the application of procedures, subjects were left to acclimatize to room conditions (23 ± 1 °C, 40–60% humidity) for 20 minutes in the seated position. Perfusion signals from LDF and PPG were simultaneously measured on the inferior aspect of the second and third toes in both feet (Fig. 1). This was a practicality-based option since not every first toe is large enough to accommodate both probes in adequate measuring conditions. In addition, large areas of skin on the first toe are frequently thick enough to alter the light penetration. We found no significant differences in perfusion measured by LDF or PPG in these conditions among any of the toes (data not shown). For the massage protocol, subjects were positioned supine, both legs slightly flexed, with one of the legs, randomly chosen, used as control. For the hyperoxia protocol subjects remained seated, both feet flat on the floor, knees and shoulders aligned.

#### Measurement technologies and signal processing

LDF signals were acquired with a Periflux 5000 device (Perimed, Sweden) and PF5010 units. Two small angled probes were used, placed with double-sided adhesive strips (PF105–3, Perimed, Sweden) on the toe’s inferior aspect (Fig. 1). Signals were acquired at a 32 Hz sampling rate with a time constant of 0.2. The LDF probe had a 0.25 mm fiber separation and emitted a laser beam of 780 nm wavelength.

PPG signals were acquired by reflection photoplethysmography (PPG) with a Blood Volume Pulse (BVP) sensor attached to a BITalino plugged microprocessor board (PLUX Wireless Biosignals, Portugal). The sensor emitted a green light of 530 nm wavelength (2.3 mm separation between LED and receptor) and was adhered to the inferior aspect of the toe using a double-sided adhesive strip (Jurasstic High Bond Tape, UK). PPG signals were acquired with a 100 Hz sampling rate, and were pre-smoothed by a moving average filter here described as:

1 $${y}_{s}(i)=\frac{1}{2N+1}((y(i+N)+y(i+N-1)+\ldots +y(i-N))$$

where *y*_*s*_(*i*) is the smoothed value for the i-th data point, N is the number of neighboring data points on either side of *y*_*s*_(*i*) and 2 N + 1 refers to the data span. Signals were then down-sampled to 32 Hz before further processing.

The Wavelet Transform (WT), a well-known tool for refined spectral analysis^22^, was applied to all signals to assess their main activity components. For each signal, a mean spectrum of all phases was calculated for each protocol. For Group 1, the mean LDF and PPG spectra from the massaged foot were compared with the ones from the control foot. In Group 2, LDF and PPG spectra are mean values from both feet.

PPG spectra were found to be similar to LDF, showing closely related frequency ranges for all components, in line with other reports^28,47^. From these frequency spectra, the frequency range, obtained by visual analysis, kurtosis and skewness of each spectral band were also calculated. Signal pre-processing and WT analysis were performed using Matlab software (Mathworks R012, USA).

#### Challenge procedures

Massage: After acclimatization to the laboratory environmental conditions, Group I individuals were submitted to a massage protocol (“effleurage”) performed by a specialized therapist. This reference technique^31,51^ was applied to a randomly chosen leg in the upwards direction (from the ankle towards the knee), and consisted of the application of a series of light compression strokes with the palms of both hands along the leg’s longitudinal axis, in a rhythmic and repetitive way. Almond oil was used as a lubricant to reduce discomfort^52^. The contralateral leg remained in the same position and was used as control. The protocol included three phases – a 10 min baseline resting register (Phase I), a 5 min massage challenge (Phase II), and a 10 min recovery (Phase III).

Hyperoxia: After acclimatization to the laboratory environmental conditions, a special facemask (AGA MedControl 45 Bar Oxygen Demand Valve facemask, AGA AB, Sweden) was applied to Group 2 subjects to guarantee a fully controlled breathing with a 100% oxygen (Medical oxygen, AirLiquide, PT) saturated atmosphere. The protocol included three phases – a 10 min baseline resting register with the volunteer breathing room atmosphere (Phase I), after applying the mask, a 10 min challenge by breathing a 100% oxygen atmosphere (Phase II), and a 10 min recovery (Phase III) again breathing room atmosphere^37^.

### Variables and statistics

For each phase of both protocols, perfusion changes were calculated as the mean value of the LDF signal and as the mean amplitude of the PPG signal, both expressed in arbitrary units (AU). For the component analysis, the activity of each component was calculated as the percent ratio of the area under the curve (AUC) of that component on the average signal frequency spectrum (30 minutes) divided by the AUC of the entire frequency spectrum. For statistical purposes, the components’ activities were calculated in three analysis periods selected inside the cone of influence (COI) of the WT scalogram. For the massage protocol, 4.00 min to 9.00 min corresponds to Phase I, 12.00 min to 15.00 min corresponds to Phase II and 17.00 min to 25.00 min corresponds to Phase III. For the hyperoxia protocol, 4.00 min to 9.00 min corresponds to Phase I, 12.00 min to 17.00 min corresponds to Phase II and 21.00 min to 26.00 min corresponds to Phase III. To assess statistical equivalency between subjects at baseline, the mean perfusion and the components’ activities were compared between the different subjects with the Wilcoxon signed rank test for one sample. To assess physiological changes in the signals’ components with each challenge, the mean perfusion and the components’ activities were compared between phases with the Wilcoxon signed rank test for paired samples. For the massage protocol, these variables were compared between massaged and control feet by the Mann-Whitney U test for independent samples. Linear correlations of the percent variation of LDF and PPG signals between Phases I and II were tested with Spearman’s R coefficient. All statistical tests were carried out with SPSS 22 (IBM, USA) and a p < 0.05 value adopted.

## Conclusions

LDF and PPG use different light wavelengths, with different penetration, sensitivity, and reflection capacities. Even accepting that some of the pulsatile and superimposed determinants might be the same, it is not granted that each frequency component in LDF and PPG have the same origin. In fact, the not so obvious correlation between both spectral profiles obtained under the same conditions reinforces that view, meaning that the oscillations registered with both signals are different. PPG revealed higher depth discrimination than LDF to detect hemodynamically-imposed changes in the contribution of the different components, irrespective of the challenge, which seems to be an additional value to explore. Furthermore, the simultaneous use of LDF and PPG offers a complementary view on the observed perfusion events, where the WT spectral signal decomposition greatly improves their quantification and opens new mechanistic directions of skin microcirculatory flowmotion to explore.
